# Adsorption–desorption behavior of the endocrine-disrupting chemical quinestrol in soils

**DOI:** 10.1038/s41598-020-70300-x

**Published:** 2020-08-06

**Authors:** Mingcheng Guo, Qin Lin, Zhenlan Xu, Chunrong Zhang, Xueping Zhao, Tao Tang

**Affiliations:** 1 Institute for the Control of Agrochemicals, Ministry of Agriculture and Rural Affairs, Beijing, 100125 People’s Republic of China; 2 State Key Laboratory for Quality and Safety of Agro-Products, Key Laboratory for Pesticide Residue Detection of Ministry of Agriculture and Rural Affairs, Institute of Quality and Standard for Agro-Products, Zhejiang Academy of Agricultural Sciences, NO. 298 Desheng Road, Hangzhou, 310021 People’s Republic of China

**Keywords:** Ecology, Environmental sciences

## Abstract

Quinestrol (QUN), a synthetic estrogen used as an oral contraceptive or emergency contraceptive component, has been shown to be an endocrine-disrupting chemical. To assess the environmental risk of QUN, batch equilibration experiments were conducted to investigate the adsorption–desorption of QUN in five contrasting soils from different areas of China. The leaching properties were also calculated based on the adsorption and degradation data from our previous study with the same soils. The Freundlich and Langmuir models were applied to the sorption–desorption data to examine the affinity towards QUN of the soils, which had varying physical and chemical properties. The K_f_ and K_f_^des^ values of QUN in the tested soils ranged from 3.72 to 20.47 mg^1−n^ L^n^ kg^−1^ and from 1.26 to 7.8 mg^1−n^ L^n^ kg^−1^, respectively, and *Q*_m_ ranged from 28.25 to 126.58 mg/kg. The desorption data showed that hysteresis occurred. The K_f_ and K_f_^des^ values of QUN were positively correlated with the soil total organic carbon (OC) and cation exchange capacity (CEC), and it may be due to the content of TOC and CEC exhibited a positive correlation. A low mobility potential of QUN in soils was predicted and verified the adsorption results by the groundwater ubiquity score (GUS) and retardation factor (R_f_).

## Introduction

Steroid hormones are an emerging endocrine disrupting chemicals (EDCs) that can interfere with the endocrine function in organisms at low concentrations^1^. synthetic steroid hormones have frequently been reported to demonstrate the higher affinities for binding to hormone receptors than natural steroid hormones and thus great disruption potencies^2,3^. Quinestrol (QUN) is a synthetic progestogen that has been used as an oral contraceptive and hormone replacement for women since the 1960s^4–6^. In recent years, some studies have reported on the adverse effects of QUN on male rat, crucian carp and duckweed after they enter the environment^7–9^. Moreover, previous reports demonstrated that the half-life periods of QUN in nature waters were in the range of 60–90 days, and the degradation rate for fifty percent (DT_50_) and 90% (DT_90_) in soils were approximately 15 days and 50 days, respectively^10–12^. The longer residual period of QUN in the environment indicated that QUN may exert toxic effects on organisms for quit a long time in its great disruption potencies. Thus, it is very necessary to understand the environmental fate of QUN and whether it is safe for organisms.

QUN is not efficiently eliminated by municipal treatment plants like other endocrine-disrupting chemicals. The municipal treatment plant effluents and wastewater discharges are considered to be the major source of QUN that are directly released into the environment^13^. QUN is generally generated by natural and anthropogenic processes and can be introduced into the soil through various routes, including human and animal excretion, farmland irrigation with municipal wastewater effluent and improvement in farmland fertility with sludge. QUN is the 3-cyclopentyl ether of ethinyl estradiol (EE2), After oral administration, it is stored in adipose tissue where it is gradually released and metabolized principally to EE2^14^. The potent effect of QUN is approximately several times higher than EE2 with a very long biological half-life of more than 5 days^15^. The sulfate and glucuronate conjugates of QUN and EE2 are formed in the kidney and excreted by human and livestock in the urine, and they can be desulfated and deglucuronidated by corresponding enzymes in the environment and converted back into its precursors^16^.

After entering soil, the endocrine-disrupting chemical QUN mainly undergo adsorption/desorption, leaching, biodegradation and biotransformation. In recent decades, most studies concerning the degradation, migration, transformation, and photodegradation of natural estrogens, such as estrone (E1), 17α-estradiol (αE2), 17β-estradiol (βE2), and estriol (E3), and the synthetic estrogen EE2 in soil have been investigated extensively^17–20^. Although the adverse effects caused by synthetic steroid hormones, such as QUN, have received increasing attention, only a few studies about the photodegradation, degradation, and accumulation in organisms of QUN in the environment were available when this work began^8–12^. In view of the low-dose effect of QUN as endocrine disruptors, knowledge of adsorption and desorption of QUN is urgently required because information on the soil transport is significant for accurate evaluation of its environmental risks.

To obtain this information, the batch equilibration method recommended by the United States Environmental Protection Agency was used to study the adsorption–desorption behavior of QUN in five contrasting soils from different areas of China. The Freundlich and Langmuir models were applied to fit the observed sorption–desorption data to derive kinetics and isotherms of QUN in the tested soils. Additionally, the groundwater ubiquity score (GUS) and retardation factor (R_f_) was used to calculate and verify the mobility potential of QUN in soils. This work would provide a supplement for the adsorption–desorption behavior and improve our understanding of potential environment risk concerning the endocrine-disrupting chemical QUN.

## Materials and methods

### Chemicals and reagents

QUN (purity > 99%) was obtained from Sigma-Aldrich (Dorset, U.K.). Ammonium acetate, anhydrous calcium chloride, and acetic acid were of analytical grade and purchased from Sinopharm Chemical Reagent Co., Ltd. (Beijing, China). Methanol and acetonitrile were high-performance liquid chromatography (HPLC)-grade chemicals obtained from Fisher Scientific (Fair Lawn, NJ). Ultrapure water used for the preparation of samples and mobile phases was obtained in the laboratory using a Milli-Q water purification system (Millipore, Billerica, MA) and had a resistivity greater than 18.2 MΩ cm.

### Soil samples

The tested soil samples included 4 farmland soils and 1 natural grassland soil. The farmland soils were taken from Heilongjiang Province (HLJ), Beijing (BJ), Yunnan Province (YN), and Guangxi Province (GX), China, and the grassland soil was taken from Inner Mongolia (NMG), China. Each soil sample was taken from the surface layer (0–20 cm). After the soil samples were collected, they were spread evenly in a clean laboratory. Next, the plant residues, stones and other debris were removed, and the samples were air-dried and passed through a 2 mm sieve. The content of total organic carbon (TOC) and nitrogen (N) in soil samples were determined by dry combustion with a CN analyzer Vario Max (Elementar Analysen systeme GmbH, Hanau, Germany). The cation-exchange capacity (CEC) was determined using a 1.0 mol/L ammonium acetate solution (pH 7.04). The particle size was determined by the hydrometer method^17^. Soil pH values in 0.01 M CaCl_2_ with a soil/solution ratio of 1:1 was determined with a pH meter. The treated soil samples were placed in plastic bags and stored in a refrigerator at 4 °C for future use. Soil sterilization was accomplished by autoclaving the soils at 120 °C under 300 kPa 3 times for 45 min in consecutive days with 24 h intervals. The properties of these soils have been determined in a previous study of ours^10^, and these soils parameters are shown in Table 1.

**Table 1:** Properties of tested soils.

Soil site	Soil	pH	N (%)	TOC^*a*^ (%)	CEC^*b*^ (cmol/kg)	Particle size (%)		
	Textural					Sand	Silt	Clay
BJ	Loam	7.1	0.13	1.95	25.6	46. 7	37.5	15.9
NMG	Sandy loam	7.5	0.08	1.14	8.7	85.0	11.3	3.7
YN	Clay loam	6.8	0.19	1.30	12.9	6.4	63.2	30.4
HLJ	Silty loam	7.0	0.21	2.45	43.6	15.3	71.1	13.6
GX	Silty loam	6. 7	0.12	1.97	22.4	38.2	53.4	8.4

**TOC**^***a***^ = total organic carbon content. **CEC**^***b***^ = cation-exchange capacity.

### Adsorption–desorption kinetics

A batch equilibration method with parallel sampling was used, as recommended by the Test Guideline OPPTS 835.1230^21^. In the adsorption test, a 2.0 g soil sample was accurately weighed in a 100 mL glass conical flask with a grinding stopper, and 50 mL of 0.01 M CaCl_2_ solution containing 1 mg/L QUN was added, resulting in a ratio of soil to water of 1:25 (W/V). The conical flasks were placed on a vibrator and continuously vibrated in the dark. After adsorption equilibrium, centrifugation at 4,500 rpm (3,560 g) for 5 min was applied to separate the aqueous phase and solid phase with a plastic (polytetrafluoroethylene) centrifugal cup. The supernatant (10 mL) was transferred into a tube and stored in a refrigerator at − 21 °C for further extraction and analysis.

The desorption test was performed as follows. After the QUN reached adsorption equilibrium in the soil, the remaining samples in the conical flasks were transferred into 100 mL centrifuge tubes and centrifuged for 5 min at 4,500 rpm (3,560 g), and the supernatant was discarded. Then, an equal volume of 0.01 M CaCl_2_ solution was added again. After full rotational mixing, the samples were placed on an oscillator. As was the case in the desorption test, at certain intervals, the flasks were removed to collect 10 mL samples of supernatant, and the supernatants were stored in the refrigerator at − 21 °C before analysis. Two blank tests were conducted without QUN and soil. Three replications were set in each treatment.

### Adsorption–desorption isotherms

The adsorption isotherms were tested by weighing 2.0 g tested soil in a 100 mL glass conical flask with a grinding stopper and mixing in 50 mL CaCl_2_ solution with QUN concentrations ranging from 0.2 to 2 mg/L. The conical flasks were shaken for 24 h in the darkness, and then supernatant (10 mL) was transferred into a tube and stored in a refrigerator at − 21 °C for further extraction and analysis. When the different concentrations of QUN reached adsorption equilibrium in the soil, the desorption isotherms were determined. The suspensions were transferred to 100 mL centrifuge tubes, and all tubes were centrifuged at 4,000 rpm for 5 min. The supernatant was discarded, and the pellet was mixed with an equal volume of CaCl_2_ solution. After testing the samples throughout mixing, they were shaken again for 48 h. Then, supernatant (10 mL) was transferred into a tube and stored in a refrigerator at − 21 °C for further extraction and analysis.

### Sample extraction and HPLC analysis

For the adsorption step, a spiked control experiment indicated that no QUN sorption on the test vessel surfaces occurred, and a mass balance experiment (At the end of adsorption and desorption test, the concentrations of QUN were analyzed in aqueous phase and soil respectively to verify whether the biodegradation of QUN occurred all along the adsorption–desorption experiment) demonstrated that no abiotic degradation occurred. Therefore, only the aqueous phase was analyzed, as recommended in the U.S. EPA Test Guideline. The aqueous sample was transferred to a 60 mL separatory funnel with 10 mL of ethyl acetate and shaken for 5 min on a mechanical shaker. Then, the solvent was collected, and the aqueous sample was extracted again. Finally, the solvent extracts were combined and evaporated to dryness on a vacuum rotary evaporator at 45 °C. The residue was reconstituted with 1 mL of HPLC-grade methanol and used for analysis by HPLC.

The analysis of QUN was performed using the HPLC method coupled with an ultraviolet–visible detector according to our previous report^12^. Preliminary experiments showed that the limit of quantification (LOQ) was 10 μg/L for aqueous samples. External calibration curves were generated to estimate the sample concentrations from peak areas. The percent recovery of QUN was higher than 95% with relative standard deviations of 2.8–4.6% for the adsorption experiment.

### Data analysis

The adsorbed concentration of QUN in the soil was calculated by the following equation:

$$C_{s} = { 5}0 \, \left( {C_{0} - C_{aq} } \right)/{2}$$

where *C*_*aq*_ (mg/L) is the concentration of QUN in the water after adsorption by the soil and *C*_*s*_ (mg/kg) is the residual QUN in the soil.

The formula for calculating the residual after the QUN in the soil reached desorption equilibrium was as follows:

$$C_{s}^{des} = \, \left( {{2}C_{s} - {5}0C_{aq}^{des} } \right)/{2}$$

where *C*_*aq*_^*des*^ (mg/L) and *C*_*s*_^*des*^ (mg/kg) are the concentrations of QUN after desorption in the water and soil, respectively.

The expression of the Freundlich equation is as follows:

$$\begin{aligned} C_{{\text{s}}} = & {\text{K}}_{{\text{f}}} C_{{{{\rm aq}}}}^{{\text{N}}} \\ C_{{\text{s}}}^{{{\text{des}}}} = & {\text{K}}_{{\text{f}}}^{{{\text{des}}}} C_{{{{\rm aq}}}}^{{{\text{desNdes}}}} \end{aligned}$$

where K_f_ (mg^1−n^ L^n^ kg^−1^) and K_f_^des^ are the adsorption and desorption constants, respectively, and N and N_des_ are constants that are related to the nonuniformity of the soil surface.

The Langmuir equation is provided by the following:

$${1}/C_{s} = { 1}/{\text{Q}}_{{\text{m}}} + { 1}/{\text{K}}_{{\text{L}}} {\text{Q}}_{{\text{m}}} C_{{{{\rm aq}}}}$$

Q_m_ (mg/kg) was the maximum adsorption concentration of QUN in the soil. K_L_ (L/mg) is the constant of the Langmuir equation, which is related to the adsorption bond energy.

The sorption and desorption data were fit by Freundlich and Langmuir models that were calculated using Sigma Plot, version 13.0. The analysis of variation (ANOVA) and regression analysis were calculated using SPSS, version 17.0.

## Results and discussion

### Adsorption–desorption kinetics of QUN in soils

**Figure 1:**
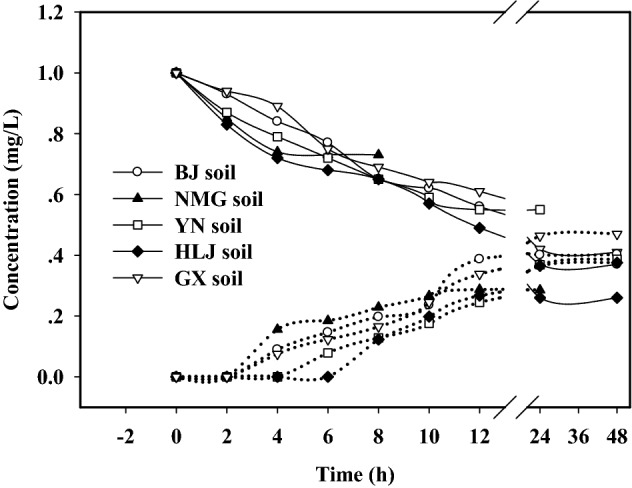
Adsorption–desorption kinetics of QUN in soils.

The adsorption–desorption kinetic experiment was designed to evaluate the minimum time of equilibrium for the tested soils. Figure 1 shows the results of QUN adsorption and desorption as a function of time for 5 selected soils. The adsorption kinetics of QUN in soils consisted of two steps: a fast phase and a slow phase. In the early stage, QUN adsorption occurred as a rapid reaction, showing a sharp decrease in solution concentration, around 12 h, the concentration of QUN decreased slightly into balance. The adsorption equilibrium time of QUN in the soil sample from NMG was the shortest, only 4 h, followed by 6 h in Yunnan soil, 8 h in Beijing soil and Guangxi soil, and 12 h in Heilongjiang soil. This phenomenon was likely due to the fact that the vacant sites in NMG soil (sand) with less contents of TOC (1.14%) were fully exposed and easily filled up, and the vacant sites of other soils were slowly occupied because of the competition between solute molecules and soil cations. This trend is similar to the kinetics of adsorption of estrogen^22,23^. To ensure the adequacy of adsorption equilibrium and the accuracy of the results, the adsorption equilibrium time of QUN in all soil samples was set to 24 h.

The time to reach desorption equilibrium of QUN was slower than the time to reach adsorption equilibrium. The results showed that no concentration of QUN in aqueous phase was detected within 2 h of the initial stage. Until 4 h of the desorption test, the concentration of QUN in aqueous phase was just detected. The desorption equilibrium time of QUN in the five tested soils varied widely: 6 h in NMG soil, 12 h in Yunnan and Guangxi soils, 24 h in Beijing soil, and 48 h in Heilongjiang soil with the highest TOC contents (2.45%). The phenomenon indicated that TOC content maybe the main factor controlling desorption of QUN in soils. In fact, the desorption of QUN in our study was ordered according to the TOC content of the soils. This hypothesis is supported by the previous reports, which have shown that TOC is the most important factor in the adsorption and desorption of EE2^24^. Then, the test time was set for 48 h to ensure the establishment of a desorption equilibrium.

### Adsorption–desorption isotherms of QUN in soils

**Figure 2:**
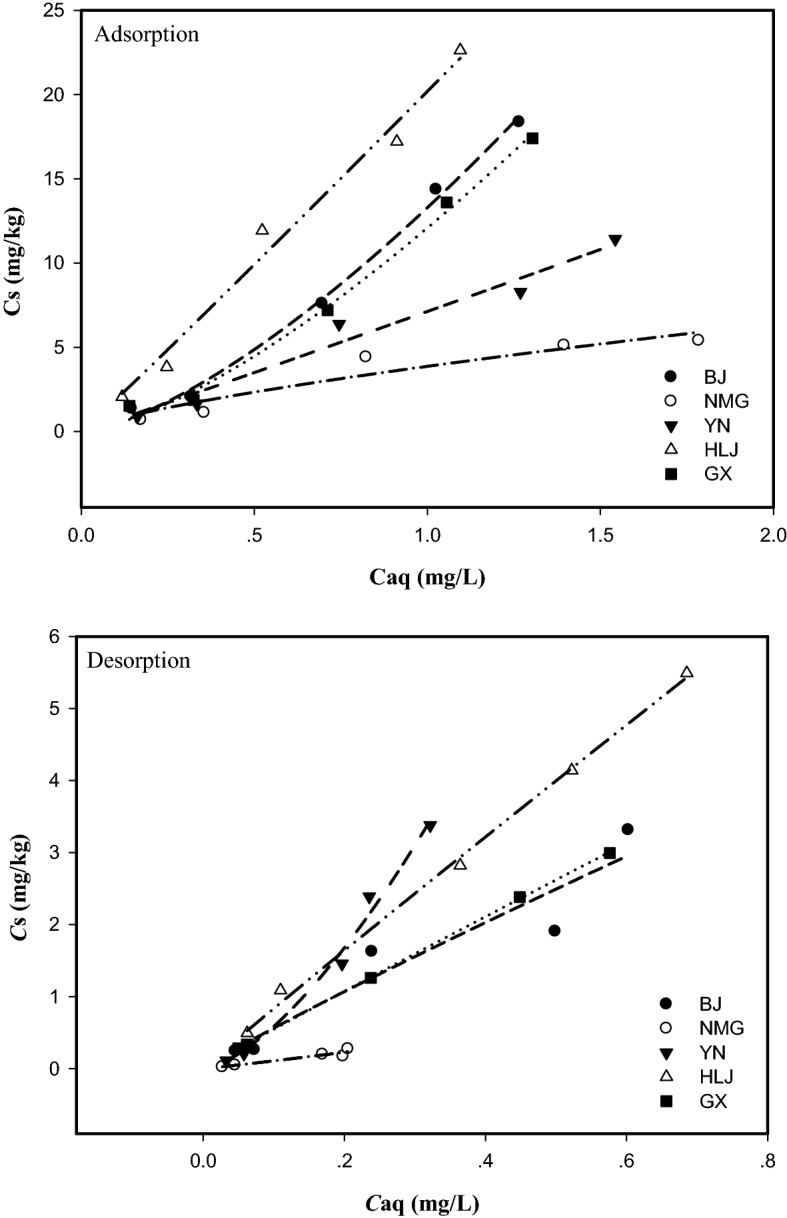
Adsorption–desorption isotherms derived from Freundlich equations for QUN in soils.

Table 2 summarizes the adsorption isotherm constants and characteristics derived from Freundlich and Langmuir equations for QUN in test soils. The data suggested that the sorption capacity of soils and their organic fractions for QUN was in the order: HLJ > BJ > GX > YN > NMG, which was in the same order of their TOC (Table 1). A similar result was reported in EE2 to natural soils and their organic fractions^24^. The values of N for soil adsorption were higher than 1, except for the sample from NMG. These adsorption curves were not linear, but with a certain curvature, and can be defined as type L^25^, and the adsorption capacity of QUN in test soils increased with increasing soil concentration. Maximum adsorption values predicted by the Langmuir equation that Q_m_ of QUN ranged from 30.86 to 126.58 mg/kg in the test soils, indicating the largest adsorption concentration in BJ soil and the lowest adsorption concentration in GX soil. K_L_ values are related to the energy of interaction between adsorbates (QUN) and adsorbents (soils) and in the current study ranged from 0.07 to 0.370. The Freundlich model fitted quite well with the measured data obtained from the sorption and desorption isotherms of test soils (Fig. 2). The adsorption behavior of QUN in the soil samples satisfied the Freundlich equation, which had correlation coefficients greater than 0.983.

**Table 2:** Adsorption isotherm constants and characteristics derived from Freundlich and Langmuir equations for QUN in the five types of soil.

Soil site	Freundlich model			K_oc_^*a*^ (L/kg)	ΔG^*b*^ (KJ/mol)	Langmuir model		
	K_f_ (mg^1−n^ L^n^ kg^−1^)	N	R^2^			K_L_	Q_m_ (mg/kg)	R^2^
BJ	12.64	1.268	0.952	648.21	− 27.67	0.070	126.58	0.918
NMG	3.72	0.959	0.926	326.32	− 24.73	0.114	34.97	0.958
YN	6.95	1.148	0.970	534.62	− 26.84	0.174	30.86	0.980
HLJ	20.47	1.098	0.982	835.51	− 28.75	0.159	105.26	0.987
GX	11.33	1.171	0.905	575.13	− 27.16	0.370	28.25	0.913

**K**_**oc**_^***a***^ = 100 K_f_ /TOC (%). **ΔG**^***b***^ =—1.724RTlnKoc.

The Koc values of QUN ranged from 326.32 to 835.51 L/kg. Generally, for a given organic compound, the value of Koc in different soils is essentially constant. In this study, the K_f_ values of QUN in the five soils varied by a factor of 5.5, and the K_oc_ values varied by a factor of 2.6. Although the variation in QUN sorption decreased significantly after the Koc value was fixed, the range of values was still wide, which suggested that the soil adsorption of QUN is controlled by other factors besides OC content. Hildebrand et al. found values of Kf and Koc for EE2 in four different soils of 28–121 ng^1−1/n^ g^−1^ mL^1/n^ and 1,127–3,941 mL/g, respectively, and soils containing more soil OC content and clay content sorbed more EE_2_, but the effect on the process of desorption was minimal^26^.

The free energy change for adsorption can be used as a preliminary indicator of the soil adsorption mechanism. According to the change in the free energy of adsorption, the process of the adsorption reaction can be inferred. When the absolute value of G is less than 40 kJ/mol, the process belongs to physical adsorption, the adsorption equilibrium of compounds in the soil is fast, and the process is reversible. When the absolute value of G is greater than 40 kJ/mol, the process belongs to chemical adsorption, the adsorption equilibrium is slow, and the adsorption process is irreversible^27^. Based on the Koc value, the free energy of QUN adsorption was calculated by the following formula:

$$\Delta {\text{G }} = \, - { 1}.{\text{724RTlnK}}_{{{{\rm oc}}}}$$

The ΔG of QUN in the soils was between − 28.75 and − 24.73 kJ/mol, and the absolute values were less than 40 kJ/mol, which demonstrated that the absorption of QUN in soils corresponded to physical adsorption.

The related Freundlich isotherm parameters for the desorption of QUN are presented in Table 3. The values of K_f_^des^ ranged from 1.26 to 7.80 mg^1−n^ L^n^ kg^−1^ with R^2^ in the range 0.935–0.999, and the hysteresis coefficient (H) values between 0.75 and 1.24. Hysteresis phenomena occur when desorption isotherms do not coincide with adsorption isotherms, which is commonly observed in chemical pollutants in soils. Theoretically, there is no hysteresis when H = 1, but in practice no hysteresis is considered when H lies between 0.7 and 1^28^. The hysteresis coefficient (H) values in soils from BJ, HLJ and GX were slightly greater than 1, indicating that the desorption of QUN in these soils was slightly hysteretic. In general, when chemical compounds are transferred to soil, their molecules can form stable bonds with soil colloids, especially organic matter and clay minerals^29^. Durán–Álvarez et al. proposed that the hysteresis of estrone and 17β-estradiol was primarily caused by the quantity of organic matter in the soil. The higher the content of organic matter in the soil is, the lower the desorption effect^30^. In addition to binding hysteresis, hysteresis is associated with fractions of soil that undergo structural hysteresis^31^. Hence, an obvious desorption hysteresis phenomenon can easily form. The results of this study were consistent with this conclusion.

**Table 3:** Freundlich parameters and hysteresis coefficient for desorption of QUN.

Parameters	Soil sites				
	BJ	NMG	YN	HLJ	GX
K_f_^des^	5.07	1.26	5.95	7.80	5.13
N_des_	1.0189	1.0680	1.5403	0.9590	0.9715
R^2^	0.935	0.967	0.992	0.988	0.999
H = N/N_des_	1.24	0.90	0.75	1.14	1.21

### Correlation between K_f_/K_f_^des^ and soil physical and chemical properties

**Figure 3:**
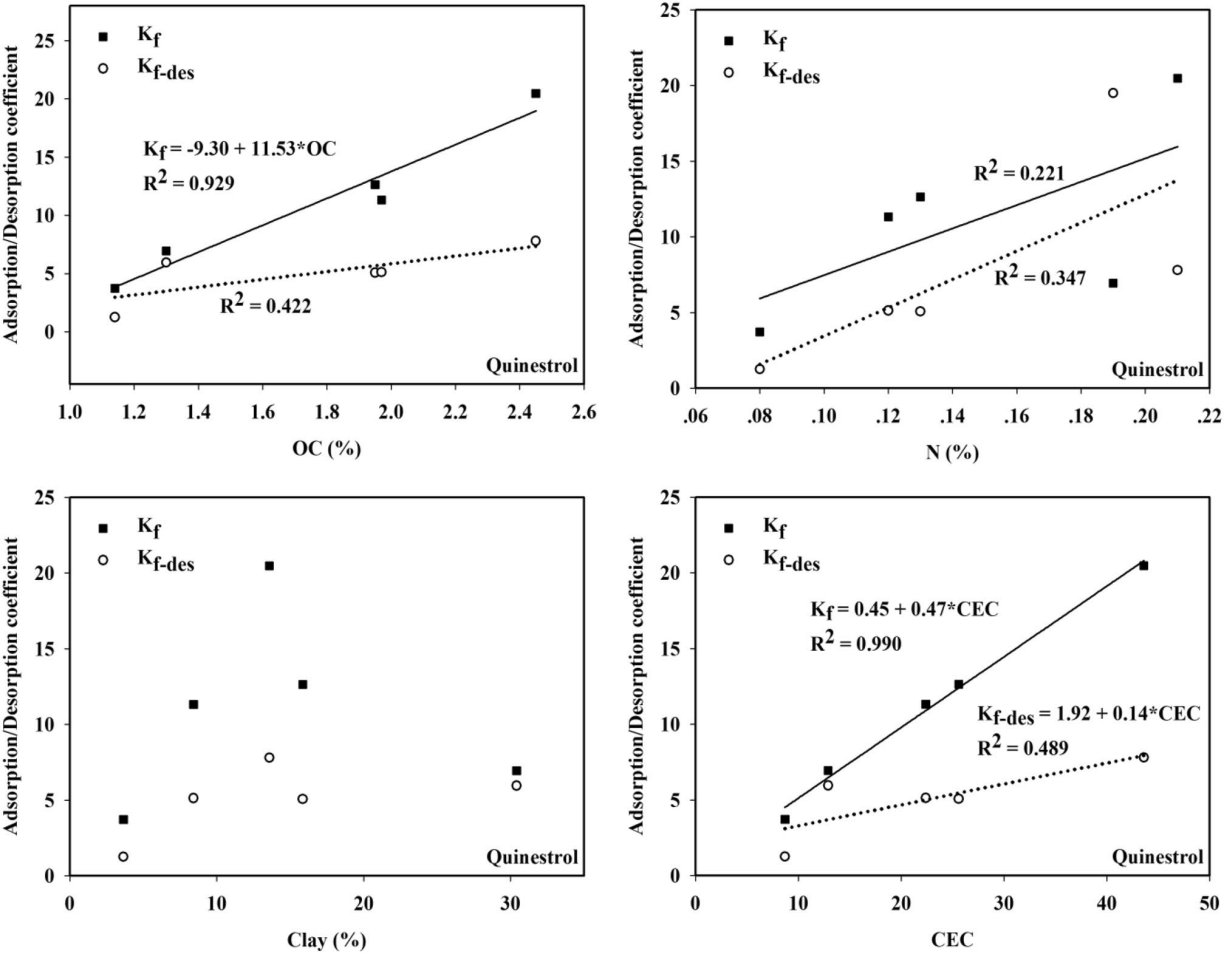
Correlations between K_f_/K_fdes_ and parameters of soil for QUN.

In addition to the properties of the compound of interest, the adsorption capacity of a compound in soil is mainly related to the physical and chemical properties of the soil, such as the soil pH, OC content, nitrogen content, CEC and clay content^32^. Based on the data, we found that the numerical values of the adsorption coefficient K_f_ and desorption coefficient K_f_^des^ varied greatly, which indicated that the adsorption and desorption capacities of QUN were obviously different and were possibly influenced by the differences in physical and chemical properties in the soils. Figure 3 shows the relationship between K_f_/K_f_^des^ and the soil physical and chemical properties. The equation relating K_f_ and soil TOC content was K_f_ = − 9.30 + 11.53 TOC, with an R^2^ of 0.929 (*p* < 0.05). The CEC was related to K_f_ by K_f_ = 0.45 + 0.47CEC, with an R^2^ of 0.990 (*p* < 0.01). An R^2^ of 0.221 was obtained between K_f_ and the soil total nitrogen content, but K_f_ was not associated with clay content, which means that the adsorption capacity of QUN has a positive relationship with OC and CEC. The R^2^ between K_f_^des^ and soil OC was 0.422, and the R^2^ between K_f_^des^ and CEC was 0.489, with a correlation equation of K_f_^des^ = 1.92 + 0.14 CEC, which indicated that the QUN desorption capacity had a positive relationship with CEC. In addition, the R^2^ for the relationship between K_f_^des^ and the soil total nitrogen content was 0.347, but K_f_^des^ was not related to the clay content. The results showed that the adsorption and desorption characteristics of QUN in soil were mainly controlled by the soil OC content and CEC, but were poorly correlated with other soil properties. For the synthetic estrogen EE_2_, K_f_ increased with increased carbon concentration with the relationship K_f_ = − 1.222 + 0.381 OC and R^2^ = 0.922, but the trend of K_f_^des^ was opposite to that of K_f_, where K_f_^des^ = 46.382–4.371 OC, with R^2^ = 0.999^31^. Durán-Álvarez et al. found that irrigating soil with wastewater containing organic matter influenced the absorption of hormones in the soil^30^.

### Leaching characteristics of QUN in soil

Based on the values of K_f_ and the degradation half-life of QUN in soil from our previous report^12^, the leaching characteristics were estimated by the groundwater ubiquity score (GUS)^33^ and retardation factor (R_f_)^34^, which are listed in Table 4.

**Table 4:** Retardation factors and groundwater ubiquity score of QUN in soils.

Soil site	Soil property		Retardation factor	GUS	
	Bulk density, ρ_b_	Porosity^a^, θ	R_f_ = 1 + ρ_b_K_f_/θ	DT_50_^b^	Log(DT_50_) × [4 − log (K_OC_)]
BJ	1.36	0.49	36.08	9.44	1.16
NMG	1.65	0.38	17.15	14.72	1.74
YN	1.29	0.51	18.58	13.12	1.42
HLJ	1.48	0.44	69.85	9.92	1.07
GX	1.33	0.50	31.13	9.83	1.23

^a^Soil porosity = 1 − (soil bulk density/2.65).

^b^DT_50_ values from our previous report^16^.

The R_f_ of QUN in the tested soils was between 17.15 and 69.85, where the R_f_ of HLJ soil was the maximum, and the R_f_ of NMG soil was the minimum; these results are in good agreement with the soil OC content and the adsorption capacity of QUN in these two types of soils. GUS has been widely used in the field of pollutants and is a useful index to describe the leachability of compounds in soil. When GUS < 1.8 in soil, compounds are considered to undergo little leaching and migration; when 1.8 < GUS < 2.8, it indicates the possibility of leaching under certain conditions; and when GUS > 2.8, it means that compounds have high leaching mobility^35^. In this study, the GUS of QUN in soil was less than 1.8, suggesting that QUN is a low-leaching substance in soil. However, the leaching characteristics of compounds in the field are complex. In addition to the properties of the compounds themselves, the leaching characteristics are related to the permeability, water content, uniformity, texture and mineral content of the soil. Therefore, the assessment of the leachability of QUN in the environment needs to take all factors into account.

## Conclusions

In this research, five contrasting soils from different areas of China were used to test the adsorption and desorption behavior of QUN, and the GUS and R_f_ were calculated and verified the leaching characteristics of QUN. The results showed that the Freundlich and Langmuir models fitted well the observed sorption–desorption data to derive kinetics and isotherms of QUN in the tested soils, and QUN could be adsorbed strongly in agricultural and grassland soils. The desorption equilibrium time of QUN was slower than the adsorption equilibrium time, and there was hysteresis in the desorption process. Binding to soil TOC appears to be the dominant sorption and desorption mechanism. The predicted GUS and R_f_ values were also verified the low mobility of QUN in tested soils. In view of the low-dose effects of QUN and its great disruption potencies, further study ought to be conducted on its distribution, transfer and dissipation in water–sediment systems.
